# HIV and cardiovascular disease in sub-Saharan Africa: Demographic and Health Survey data for 4 countries

**DOI:** 10.1186/s12889-021-11218-5

**Published:** 2021-06-12

**Authors:** Leonard E. Egede, Rebekah J. Walker, Patricia Monroe, Joni S. Williams, Jennifer A. Campbell, Aprill Z. Dawson

**Affiliations:** 1 Department of Medicine, Division of General Internal Medicine, Froedtert & The Medical College of Wisconsin, 8701 Watertown Plank Rd, Milwaukee, WI 53226-3596 USA; 2 Center for Advancing Population Science, Medical College of Wisconsin, Milwaukee, WI USA

**Keywords:** HIV, Diabetes, Hypertension, Cardiovascular disease, Demographic and Health Survey

## Abstract

**Background:**

Investigate the relationship between two common cardiovascular diseases and HIV in adults living in sub-Saharan Africa using population data provided through the Demographic and Health Survey.

**Methods:**

Data for four sub-Saharan countries were used. All adults asked questions regarding diagnosis of HIV, diabetes, and hypertension were included in the sample totaling 5356 in Lesotho, 3294 in Namibia, 9917 in Senegal, and 1051 in South Africa. Logistic models were run for each country separately, with self-reported diabetes as the first outcome and self-reported hypertension as the second outcome and HIV status as the primary independent variable. Models were adjusted for age, gender, rural/urban residence and BMI. Complex survey design allowed weighting to the population.

**Results:**

Prevalence of self-reported diabetes ranged from 3.8% in Namibia to 0.5% in Senegal. Prevalence of self-reported hypertension ranged from 22.9% in Namibia to 0.6% in Senegal. In unadjusted models, individuals with HIV in Lesotho were 2 times more likely to have self-reported diabetes (OR = 2.01, 95% CI 1.08–3.73), however the relationship lost significance after adjustment. Individuals with HIV were less likely to have self-reported diabetes after adjustment in Namibia (OR = 0.29, 95% CI 0.12–0.72) and less likely to have self-reported hypertension after adjustment in Lesotho (OR = 0.63, 95% CI 0.47–0.83). Relationships were not significant for Senegal or South Africa.

**Discussion:**

HIV did not serve as a risk factor for self-reported cardiovascular disease in sub-Saharan Africa during the years included in this study. However, given the growing prevalence of diabetes and hypertension in the region, and the high prevalence of undiagnosed cardiovascular disease, it will be important to continue to track and monitor cardiovascular disease at the population level and in individuals with and without HIV.

**Conclusions:**

The odds of self-reported diabetes in individuals with HIV was high in Lesotho and low in Namibia, while the odds of self-reported hypertension in individuals with HIV was low across all 4 countries included in this study. Programs are needed to target individuals that need to manage multiple diseases at once and should consider increasing access to cardiovascular disease management programs for older adults, individuals with high BMI, women, and those living in urban settings.

## Introduction

Globally, 38 million people were living with human immunodeficiency virus (HIV) at the end of 2019 [1]. The concentration of individuals living with HIV is most noticeable in low- and middle-income countries. For example, more than two-thirds of those living with HIV are in sub-Saharan Africa [2]. The number of individuals who have access to antiretroviral therapy (ART) has increased rapidly, resulting in reductions in AIDS-related deaths by 60% since 2004 [1]. At this time, most countries in sub-Saharan Africa have adopted “treat all” national policies for people living with HIV to ensure timely ART therapy [3]. Largely due to this expansion in treatment, the projected number of individuals living with HIV who are 50 years or older is expected to triple by 2040 in sub-Saharan Africa [4]. As individuals with HIV live longer, comorbidities become increasingly challenging for healthcare systems in low-income countries, especially Africa, to address effectively [5]. As a result, a shift in focus from communicable to non-communicable disease management for those living with HIV has occurred over the past several years [6].

In total, disability adjusted life years (DALYs) attributable to non-communicable diseases increased by 67% between 1990 and 2017 [7]. According to the Global Burden of Diseases, Injuries, and Risk Factors Study, cardiovascular diseases were the second leading non-communicable disease with changes in all-age total DALYs of 51.1 and 126.4% for hypertension and diabetes respectively [6, 7]. Longer life associated with more effective treatment, use of certain antiretroviral medications, and changes in living environments due to economic growth have all been noted as possible reasons for the growing burden of non-communicable diseases in individuals with HIV [8–21]. Urbanization, in particular, has been noted as an important change in sub-Saharan Africa [22]. Urbanization is characterized by a decrease in physical activity and an increase in high caloric food intake, thus being associated with increased risk for non-communicable diseases [22, 23]. This rate is high within sub-Saharan Africa at an annual rate of change of 3%, compared to 0.96% for the United States [22].

Uncertainties surrounding estimates of disease and disease burden across sub-Saharan Africa necessitates an adjustment in research, data collection, health systems structure [4, 7, 8, 24, 25]. Hypertension is estimated to affect 1.13 billion people, two-thirds of which live in low- and middle-income countries [26]. It is also currently the most prevalent non-communicable disease found for patients living with HIV, especially for those 40 years or older [11, 13, 17, 20, 27–37]. A systematic review of published articles between 2000 and 2017 found hypertension prevalence among those living with HIV to range between 6 and 22% across SSA [15]. Country-specific studies published separately provided prevalence data for those living with HIV and who also had hypertension ranging between 10.2 to 29.9% [10–13, 17, 19, 20, 27–43]. Undiagnosed cases were even higher, ranging from 11.2% in Zimbabwe [11], 13.3% in rural Kenya [28], 60.3% in Malawi [32], and 75% in Ethiopia [12]. Diabetes has also been noted as having a high prevalence in those with HIV, estimated at between 16 and 25% of those living in sub-Saharan Africa with HIV [15]. The prevalence of diabetes was documented in 12 countries, ranging from 2.0 to 13.2% with most common risk factors including age, BMI, and male sex [10, 16, 17, 20, 29, 30, 32, 35, 40, 44–47]. However, concern remains regarding high levels of undiagnosed diabetes, particularly in low- and middle-income countries [24].

Much of the current literature in this area focuses on single country analysis in a select number of countries, small sample sizes, populations primarily 50 years or older, and use of local or regional data, limiting the generalizability of results [15, 48]. We sought to address these limitations by utilizing a large, nationally representative data set available for a number of countries - the Demographic and Health Survey (DHS). Though DHS publishes estimates for HIV, diabetes, and hypertension independently, they do not investigate co-occurrence or report the odds for individual cardiovascular diseases based on HIV status. Therefore, the aim of this paper is to investigate the relationship between two common cardiovascular diseases (diabetes and hypertension) and HIV in adults living in sub-Saharan Africa using population data.

## Methods

### Data source and sample

Data from the nationally representative household surveys collected by the Demographic and Health Survey (DHS) program were used for this study [49]. Standard DHS surveys are collected from between 5000 and 30,000 households in countries around the world, with key performance monitoring data collected using a population based clustering by urban or rural regions to allow systematic sampling from the national sample frame for each country [49]. Approval to use DHS data was obtained from the DHS program prior to data download. No further ethics approval is required as data are de-identified and publicly available for use after approval.

Four sub-Saharan countries asked about both diagnosis of cardiovascular disease (diabetes and hypertension) and HIV when collecting Demographic and Health Survey Household questionnaires available at the time of analysis were included in the study. All adults asked questions regarding HIV, diabetes, and hypertension status were included in the analysis. Therefore, data was included for 5356 adults in Lesotho from the year 2014, 3294 adults in Namibia from the year 2013, 9917 adults in Senegal from the years 2010–2011, and 1051 adults in South Africa from the year 2016. Sampling weights, cluster, and strata information provided in each country specific file was used to account for complex survey design and allow weighting to the population.

### HIV status

HIV status served as the primary independent variable for all analyses. Blood was collected by the DHS program team from men and women between the ages of 15–49 years to determine HIV status as positive or negative [50]. Blood spots are collected using a finger prick and transported to a laboratory for testing using an initial ELISA test. Retest of 5–10% of negative tests was performed with a second ELISA with Western Blot performed on discordant results. Testing was voluntary, however, analysis by the DHS program on non-response indicated that HIV testing showed minimal bias [50].

### Cardiovascular disease status

Diabetes and hypertension status served as primary outcomes for analyses. Each was based on self-report by the respondent using the questions ‘Has a doctor or other health professional ever told you that you had diabetes’ and ‘Have you ever been told by a doctor or other health worker that you had hypertension or high blood pressure?’

### Covariates

Adjusted models included the covariates of age, gender, rural/urban residence, and body mass index (BMI). Age was treated as a continuous variable. Gender was self-reported as male or female. Location of residence was based on DHS program determination of either urban or rural residence. BMI was based on height and weight as measured by the DHS program during questionnaire completion.

### Statistical analysis

Sample characteristics for each country were calculated using descriptive statistics. A series of logistic models were run to investigate the relationship between HIV and self-reported cardiovascular disease status. Models were run for each country separately, with self-reported diabetes as the first outcome and self-reported hypertension as the second outcome. HIV status served as the primary independent variable for all models and was run first in unadjusted analyses, and secondly adjusted for age, gender, rural/urban residence, and BMI. Statistical analyses were adjusted for complex survey design using survey procedures in Stata v.14. Statistical significance was based on *p* < 0.05.

## Results

Table 1 provides information on general demographics of samples from each country and prevalence of HIV, self-reported diabetes, and self-reported hypertension in each country. Prevalence of HIV was 24.9% in Lesotho, 21.0% in South Africa, 14.3% in Namibia, and 0.7% in Senegal. Prevalence of self-reported diabetes was 3.8% in Namibia, 1.7% in South Africa, 1.2% in Lesotho, and 0.5% in Senegal. Prevalence of self-reported hypertension was 22.9% in Namibia, 15.3% in Lesotho, 9.8% in South Africa, and 0.6% in Senegal.

**Table 1:** Sample Characteristics for each Sub-Saharan African Country in Analysis

	Lesotho ***n*** = 5356	Namibia ***n*** = 3294	Senegal ***n*** = 9917	South Africa ***n*** = 1051
Mean Age	29.0 ± 0.2	32.0 ± 0.2	29.0 ± 0.2	31.1 ± 0.2
Sex				
Male	47.9%	46.5%	46.3%	50.5%
Female	52.1%	53.5%	53.7%	49.5%
Residence				
Rural	65.2%	45.4%	48.1%	32.0%
Urban	34.8%	54.6%	51.9%	68.0%
Mean BMI	23.71 ± 0.1	22.9 ± 0.1	21.7 ± 0.1	25.9 ± 0.2
Prevalence of HIV	24.9% (23.3, 26.5)	14.3% (13.2, 15.6)	0.7% (0.5, 0.01)	21.0% (19.1, 23.0)
Prevalence of Self-reported Diabetes	1.2% (0.1, 1.6)	3.8% (0.03, 0.05)	0.5% (0.4, 0.8)	1.7% (1.2, 2.2)
Prevalence of Self-reported Hypertension	15.3% (13.9, 16.9)	22.9% (20.9, 25.1)	0.6% (0.5, 0.7)	9.8% (8.6, 11.2)

Table 2 provides results from unadjusted models and Table 3 provides results from adjusted models. Individuals with HIV in Lesotho were 2 times more likely to have self-reported diabetes (OR = 2.01, 95% CI 1.08, 3.73), however the relationship lost significance after adjustment. Individuals with HIV in Namibia were 71% less likely to have self-reported diabetes after adjustment than those without HIV (OR = 0.29, 95% CI 0.12, 0.72). While individuals with HIV in Namibia were less likely to have self-reported hypertension in unadjusted models (OR = 0.65, 95% CI 0.47, 0.88), after adjustment only the relationship in Lesotho remained significant (OR = 0.63, 95% CI 0.47, 0.83). Relationships were not significant for Senegal or South Africa in unadjusted or adjusted models.

**Table 2:** Relationship between HIV and Cardiovascular Disease in Sub-Saharan Africa

	Lesotho	Namibia	Senegal	South Africa
Odds of Self-reported Diabetes				
HIV Unadjusted	**2.01 (1.08, 3.73)**	**0.19 (0.09, 0.41)**	1.24 (0.16, 9.31)	1.13 (0.53, 2.41)
Odds of Self-reported Hypertension				
HIV Unadjusted	0.81 (0.61, 1.06)	**0.65 (0.47, 0.88)**	0.75 (0.26, 2.16)	1.09 (0.78, 1.50)

Bold indicates significance at the *p* < 0.05 level

**Table 3:** Adjusted Relationship between HIV and Cardiovascular Disease in Sub-Saharan Africa

	Lesotho	Namibia	Senegal	South Africa
Odds of Self-reported Diabetes				
HIV	1.30 (0.67, 2.50)	**0.29 (0.12, 0.72)**	0.61 (0.08, 4.72)	1.02 (0.45, 2.31)
Age	**1.09 (1.05, 1.23)**	**1.08 (1.05, 1.11)**	**1.17 (1.12, 1.23)**	**1.11 (1.08, 1.14)**
Sex (Male ref)	1.51 (0.67, 3.39)	0.72 (0.44, 1.18)	2.19 (0.87, 5.51)	1.46 (0.63, 3.41)
Residence (Urban ref)	0.68 (0.39, 1.21)	**0.33 (0.20, 0.54)**	**0.22** **(0.10, 0.49)**	0.81 (0.47, 1.39)
BMI	**1.05 (1.02, 1.07)**	**1.09 (1.05, 1.14)**	1.00 (0.98, 1.02)	**1.06 (1.02, 1.09)**
Odds of Self-reported Hypertension				
HIV	**0.63 (0.47, 0.83)**	0.99 (0.71, 1.39)	0.40 (0.13, 1.20)	0.85 (0.61, 1.19)
Age	**1.06 (1.04, 1.06)**	**1.08 (1.06, 1.10)**	**1.07 (0.13, 1.20)**	**1.09 (1.08, 1.11)**
Sex (Male ref)	**1.59 (1.12, 2.26)**	1.13 (0.87, 1.49)	**6.82 (5.01, 9.29)**	**1.43 (1.03, 1.99)**
Residence (Urban ref)	0.84 (0.67, 1.06)	**0.55 (0.43, 0.70)**	**0.76 (0.59, 0.98)**	0.94 (0.72, 1.23)
BMI	**1.07 (1.04, 1.09)**	**1.10 (1.07, 1.12)**	**1.01 (1.00, 1.02)**	**1.06 (1.03, 1.08)**

Each model adjusted for age, sex, rural/urban residence, and BMI

Bold indicates significance at the *p* < 0.05 level

## Discussion

Based on results of this study, there was country-by-country variable relationship between HIV and cardiovascular disease by country. Though individuals with HIV were more likely to have self-reported diabetes in Lesotho, this relationship was explained by age, sex, residence, and BMI. Individuals in Namibia with HIV were less likely to have self-reported diabetes, and individuals in Lesotho were less likely to have self-reported hypertension, even after adjusting for covariates. This study provides novel findings suggesting HIV did not serve as a risk factor for self-reported cardiovascular disease in Sub-Saharan Africa during the years included in this study. However, given the growing prevalence of diabetes and hypertension in the region, and the high prevalence of undiagnosed cardiovascular disease [51], it will be important to continue to track and monitor cardiovascular disease at the population level and in individuals with and without HIV.

This study adds to the existing literature by providing information on the co-occurrence of HIV and two common cardiovascular disease (diabetes and hypertension) and indicates country by country variability across Sub-Saharan Africa. Similar to other studies, risk for self-reported cardiovascular diseases for those living with HIV reflect risk factors for the general population including age, BMI, sex, and residence. Based on these results, there may be specific sub-populations that should have programs targeted to address needs. First, programs may need to be developed to target individuals with HIV in Lesotho and provide information on managing both HIV and diabetes successfully. Though integration of HIV and non-communicable disease care within low-resource countries is relatively new, success has been documented within several novel programs across Sub-Saharan Africa [52–54]. Utilizing established care programs for HIV that are culturally and locally relevant has enabled countries to address non-communicable diseases alongside HIV [53–56], resulting in increased diabetes clinical services, [54], screening and referrals [53], and a decrease in negative health behaviors [52]. Secondly, as older age and higher BMI were associated with both self-reported diabetes and hypertension across all 4 countries, community-based health education program should highlight the importance of screening for cardiovascular disease in these two populations. Third, as women were more likely to self-report hypertension than men, despite other demographic factors and HIV status, this may be a group where targeted interventions on disease management should be focused. An analysis conducted in South Africa found higher risk for women for heart disease, primarily among those with the risk factor of obesity [57]. This suggests it will be important to address both disease and risk factors when developing programs. Finally, urban residence in Namibia and Senegal was associated with both self-reported diabetes and hypertension. Providing programs in these countries on managing disease within an urban setting may be another important target for public health work. The World Health Organization provides suggested measures to address non-communicable diseases in low- and middle-income countries targeting the impacts of urbanization, including taxes on alcohol and smoking, limiting advertisements, and health promotion through community wide screenings and public awareness campaigns [58]. Other recommendations include a public health approach, similar to efforts taken to address HIV, including multiple sectors and decentralizing of health care [56, 59]. These actions could result in a rapid scale up of non-communicable disease care, simplification and standardization of treatment, and provision of health education through community and peer support [56]. Future work focusing on understanding changes over time and the influence of individual wealth and national economic development will offer additional information to guide future programs.

Though our study focused on multiple countries and used data collected to allow extrapolation to the population level, there are limitations worth noting. First, data is cross-sectional as individuals were not followed over time, and causality between HIV and cardiovascular disease cannot be discussed. Second, diabetes and hypertension status were self-reported and based on prior diagnosis. Given the high burden of undiagnosed cardiovascular diseases in sub-Saharan Africa, this may result in under-reporting in the datset and thus low estimates in this analysis. Third, as data was collected between the years of 2010 and 2016, population level changes in prevalence of HIV, diabetes, and hypertension across these countries may have changed. Finally, results may not be generalizable to other countries and analyses should be replicated with data available from multiple cultures and contexts.

## Conclusion

In conclusion, the odds of self-reported diabetes in individuals with HIV was high in Lesotho and low in Namibia, while the odds of self-reported hypertension in individuals with HIV was low across all 4 countries included in this study. Programs are needed to target individuals that need to manage multiple diseases at once and should consider increasing access to cardiovascular disease management programs for older adults, individuals with high BMI, women, and those living in urban settings.

## Data Availability

The data set supporting the conclusions of this article are available through request from the Demographic and Health Survey. Registration for datasets can be completed at: https://www.dhsprogram.com/data/new-user-registration.cfm.
